# Prevalence of childhood cancer in Canada: an analysis using 5-year, 18-year and 25-year limited-duration prevalence from the CYP-C data tool

**DOI:** 10.24095/hpcdp.45.10.03

**Published:** 2025-10

**Authors:** Katherine McKenzie, Lin Xie, Rana Khafagy, Christina Ricci, Vera Grywacheski

**Affiliations:** 1 Centre for Surveillance and Applied Research, Public Health Agency of Canada, Ottawa, Ontario, Canada; 2 Department of Pharmacy, The Hospital for Sick Children, Toronto, Ontario, Canada

**Keywords:** neoplasms, prevalence, incidence, child, medical oncology, public health surveillance, survivors of childhood cancer

## Abstract

Survivors of childhood cancers can face life-long health risks. In this study we describe the prevalence of childhood cancer in Canada by type, geographic region, year, age group and sex, using publicly available data in the Cancer in Young People in Canada (CYP-C) data tool. By 2021, 4325 people aged less than 20 years who had received a cancer diagnosis within the previous 5 years were still alive. The age-standardized 5-year prevalence increased by 12% over the past 15 years. Leukemia was the most prevalent childhood cancer. The CYP-C data tool provides comprehensive and timely public health surveillance statistics to understand the burden of childhood cancer.

**Highlights**

- By 2021, more than 1 in 1000 Canadians aged less than 20 years had received a cancer diagnosis before they were 15 years old.
- The age-standardized 5-year prevalence of Canadians surviving childhood cancer increased by 12% between 2006 and 2021.
- The online Cancer in Young People in Canada (CYP-C) data tool provides timely, accurate and accessible data about childhood cancer, including prevalence estimates, which are important for allocating resources and assessing impact.
- The CYP-C data tool allows for new comparisons by cancer type, geographic region, age, sex and year to help understand the burden of childhood cancer.

## Introduction

Capturing cancer trends in children and youth is essential for understanding cancer burden.^1^ Although rare, childhood cancers significantly impact mortality^2^ and morbidity^3^,^4^ among young people. Between 925 and 1000 children aged less than 15 years are diagnosed with cancer every year in Canada, and as of 2020, 86% had survived for 5 years.^2^ Childhood cancer remains the second leading cause of death among children in Canada aged 1to 14 years.^5^

Understanding the prevalence of childhood cancer is important for health system planning, resource allocation and assessing the impact of cancer.^6^-^8^ Childhood cancer survivors require life-long survivorship care because of therapy-related complications, known as late effects.^3^ Clinical guidelines for screening and management of late effects can improve long-term follow-up care and quality of life for childhood cancer survivors.^3^

The Cancer in Young People in Canada (CYP-C) program is a national, population-based surveillance system that serves to improve pediatric outcomes.^9^ The CYP-C program operates through a collaboration between the Public Health Agency of Canada (PHAC), the Canadian Partnership Against Cancer (CPAC) and theC^17^ Council. Data are collected from 16pediatric hematology, oncology and stem cell transplant programs in Canada.^9^,^10^

The CYP-C data tool, hosted on the Government of Canada’s Health Infobase (https://health-infobase.canada.ca/), is an online interactive tool that displays data on childhood cancer collected through the CYP-C surveillance system.^2^ The data tool is the only pan-Canadian surveillance tool dedicated to childhood cancer. It supports the Government of Canada’s Open Data initiative by providing timely, accurate and accessible data. Open Data aims to provide Canadians access to data produced, collected and used across the federal government.^11^

In 2024, the CYP-C data tool was expanded to include prevalence estimates over time by age group, sex, cancer type and geographic region. Prevalence estimates of childhood cancer were previously limited to specific provinces^5^,^12^-^14^ or points in time^6^,^15^-^17^ and were often not disaggregated by risk factors.^6^,^13^,^15^-^17^ Estimates from studies of childhood cancer survivors in the United States^4^ and the adult population in Canada^15^ suggest that the prevalence of individuals with a history of cancer, including those in remission, has increased over time, likely because of improved survival.

This study aims to present prevalence estimates of childhood cancer in Canada, stratified by cancer type, geographic region and year using data shown in the CYP-C data tool. The prevalence estimates are based on cases diagnosed between 2001 and 2020 for 5-year limited-duration prevalence (LDP) and between 1992 and 2017 for the 5-, 18- and 25-year LDPs.

## Methods

***Data sources***

The aggregated incidence and prevalence data used in this study were downloaded in March 2024 as CSV text files from the publicly available CYP-C data tool, which gathers data from two sources.

The first source groups data from all children (aged less than 15 years) presenting at one of Canada’s 16 pediatric hematology, oncology and stem cell transplant programs with a diagnosis listed in the *International Classification of Childhood Cancer, Third Edition*.^18^,^19^ Each case registered in the CYP-C is followed for up to 5years after diagnosis. The Pediatric Oncology Group of Ontario (POGO) also shares their population-based pediatric cancer registry with PHAC; Ontario data are obtained from the POGO registry to complete the CYP-C dataset. Detailed information about CYP-C and POGO data are published elsewhere.^2^,^10^,^20^,^21^

The second source is the Canadian Cancer Registry (CCR), a population-based registry that includes data reported to Statistics Canada by provincial and territorial cancer registries. The CCR collects information about primary cancer diagnoses received by residents of Canada.^22^ The Canadian Vital Statistics - Death (CVSD) database includes demographic and death information reported to Statistics Canada by provincial and territorial vital statistics registries.^23^ Statistics Canada creates and shares with PHAC a linked CCR and CVSD file (CCR–CVSD).

Population estimates for Canada and the provinces and territories are based on census data from Statistics Canada.^24^

***Statistical analysis***

The number of incident cases refers to the number of children with a new diagnosis of childhood cancer (i.e. received before age 15 years). LDP refers to the number of people diagnosed with a childhood cancer over a specific length of time (5, 18 or 25years) who are alive on a given date. Person-based cancer prevalence counts the number of individuals, rather than the number of cancers diagnosed. Each statistic is based on a single cancer per person.

We estimated 5-year LDPs based on the number of cases in the CYP-C diagnosed between 1 January 2001 and 31 December 2020 who were alive on or after 1 January 2006. To calculate 18- and 25-year LDPs, we used linked CCR–CVSD data on children aged less than 15 years diagnosed between 1 January 1992 and 31 December 2017 and alive on 1 January 2018. Data from Quebec are not included in the CCR–CVSD. Detailed methods are described elsewhere.^2^

Using the counting method, we estimated prevalence from incidence and survival data.^25^,^26^ For estimates using CYP-C data, prevalence calculations were completed in SEER*Stat software.^27^ We used the Kaplan-Meier method with monthly intervals, based on age at diagnosis, sex and cancer site, to adjust the estimate of the proportion of cases lost to follow-up. For estimates using CCR–CVSD data, individuals without a record of death were presumed to be alive by the end of each time frame.

Age-standardized prevalence proportions are presented per million and standardized to the 2011 Canadian population.

***Suppression***

To ensure confidentiality, case counts of less than 5 are suppressed. In addition, case counts were randomly rounded either up or down to a multiple of 5. Age-standardized proportions use unrounded prevalent case counts.

## Results

By 1 January 2021, 4325 people aged less than 20 years who had been diagnosed with childhood cancer in the previous 5years were still alive.

**Figure 1:**
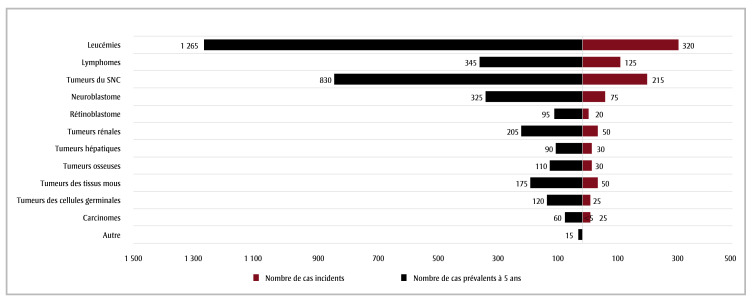
Number of incident and 5-year prevalent cases, children aged less than 15 years, by cancer type, 2020, Canada

The most frequently diagnosed childhood cancer type in 2020 was leukemia; there were 320 new cases versus 965 new cases of all cancers combined (Figure 1). Among the children aged less than 15 years living with and beyond cancer, the most common cancer diagnosis was leukemia (1265 5-year prevalent cases in 2020).

**Data source: **Cancer in Young People in Canada (CYP-C) data tool.^2^

**Abbreviation: **CNS, central nervous system. 

^a^ To ensure conf﻿identiality, counts were randomly rounded to a multiple of 5 using an unbiased random-rounding scheme. Counts may not sum to the total because of this random rounding. Counts < 5 were suppressed. 

^b^ Classified according to the *International Classification of Childhood Cancer, 3rd ed.*^19^ into 12 main groups. CNS tumours include benign and malignant tumours. 

**Figure 2:**
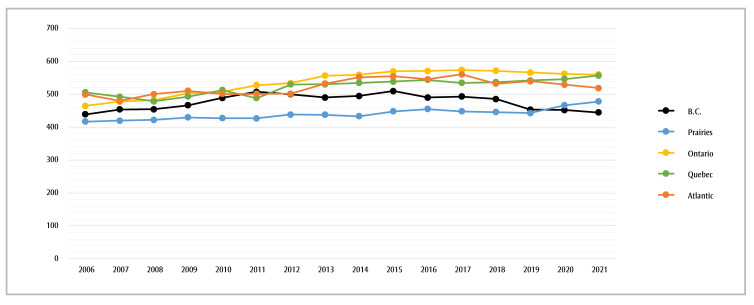
Age-standardized 5-year prevalence per 1 000 000, children and youth aged less than 20 years, by province or geographic region and year, 1 January 2006 to 1 January 2021, Canada

The age-standardized 5-year prevalence proportion in the CYP-C increased by 12% over the past 15 years, from 463 per million in 2006 to 524 per million in 2021. At 14.7% and 20.5%, respectively, this increase is most striking in the Prairies (comprising the provinces of Alberta, Saskatchewan and Manitoba) and in Ontario (Figure 2).

**Data source: **Cancer in Young People in Canada (CYP-C) data tool.^2^

**Abbreviation:** B.C., British Columbia.

^a^ Age-standardized 5-year prevalence proportions are standardized to the 2011 Canadian population.

^b^ The Atlantic region comprises the provinces of New Brunswick, Prince Edward Island, Nova Scotia, and Newfoundland and Labrador. The Prairies comprise the provinces of Alberta, Saskatchewan and Manitoba. Data from Yukon, Northwest Territories and Nunavut are statistically unstable and are suppressed.

**Figure 3:**
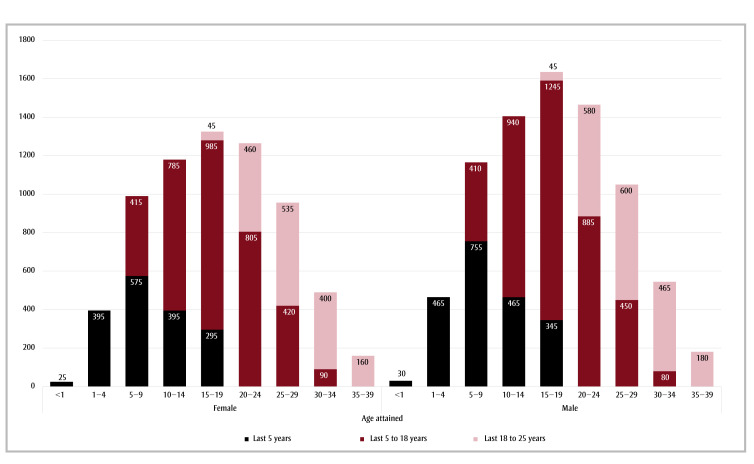
Number of 25-year prevalent cases, by sex, attained age in years and time since diagnosis, 1 January 2018, Canada (excluding Quebec)

In 2018, 8615 individuals aged less than 20 years recorded in the CCR–CVSD had received a cancer diagnosis in their lifetime; the age-standardized 25-year LDP was 1365 per million. More than 60% of those diagnosed within the 25 years prior to 1 January 2018 were more than 15 years old. Because of their higher incidence,2 males had a higher prevalence, though age distributions were similar in males and females (Figure 3).

**Data source:** Canadian Cancer Registry – Canadian Vital Statistics Death database (CCR–CVSD).^22^,^23^

^a^ To ensure confidentiality, counts were randomly rounded to a multiple of 5 using an unbiased random-rounding scheme. Counts may not sum to the total because of this random rounding. Counts < 5 were suppressed. 

## Discussion

More than 1 in 1000 Canadians aged less than 20 years had received a cancer diagnosis before the age of 15 years. Prevalence combines the number of childhood cancer patients currently receiving treatment with cancer survivors who may need survivorship care and life-long monitoring.^15^,^28^ Understanding the size of this population is vital for cancer control planning, health care resource allocation and research, and assessing impact.^1^

Our estimates are similar, although not directly comparable, to other estimates in Canada^14^-^16^,^29^ and abroad.^7^,^8^,^30^ For example, POGO^14^ and Statistics Canada29 found that by 2017 and 2018, respectively, 4700 and 4265 people living with and beyond cancer and aged less than 20 years had been diagnosed with childhood cancer in the previous 5 years. Age-standardized 5- and 20-year LDP proportions in Australia^7^ and the Netherlands,^8^ respectively, followed a similar trend to our 5- and 25-year LDPs. 

The CYP-C data tool is valuable as other Canadian estimates are not reported by pediatric cancer type or by geographical region and age at diagnosis.^14^-^16^,^29^ Proportions are similar across Canada, and the age-standardized prevalence has increased slightly over the last 15 years. Leukemia contributed to one-third of all incident and prevalent cases.

Having accessible, accurate and timely public health data is crucial for Canada.^31^ The CYP-C data tool can be used to share childhood cancer information with patients and their families, health care professionals, policy makers, advocates and researchers.

***Strengths and limitations***

The CYP-C data tool includes Canadian childhood cancer data from the two most comprehensive and timely sources. These data allow for new comparisons by cancer type, geographic region, age group, sex and time, providing the ability to look beyond incidence to understand the burden of childhood cancer.

There are some limitations. The data tool only includes LDP data of Canadians aged less than 40 years with a history of childhood cancer as the CCR does not capture data before 1992. Childhood cancer survivors aged 40 years or older are also at risk for late effects of cancer treatment.^28^ Future work will aim to include longer follow-up and complete prevalence across the lifespan to capture the full burden of childhood cancer.

When using data from the CCR, we assumed individuals to be living if there was no associated record of death. However, deaths that occur outside of Canada are not included, which could result in slightly overestimated prevalence. Also, all cases in Quebec were excluded because data sharing agreements prohibit data release.

The data tool employs suppression and unbiased random rounding to maintain privacy in public datasets.^32^ These techniques have a greater impact on small populations (e.g. small provinces or territories), which could mask important geographic variations and changes in rates over time.^33^

## Conclusion

In partnership with the CPAC and the C17 Council, PHAC recently included prevalence data of individuals living with or beyond childhood cancer in the CYP-C data tool. This study characterizes the population of childhood cancer patients and survivors. The aim is to add more data, such as socioeconomic status, in order to improve public health surveillance in this population in the future.

## Acknowledgements

The contributions of study participants, participating pediatric oncology centres, members of the CYP-C management and steering committees, POGO and its five hospital partners, the C^17^ Council, CPAC and Statistics Canada are gratefully acknowledged.

We would also like to acknowledge the contributions of several individuals. We thank Jay Onysko from the Applied Research Division at PHAC and Mylene Frechette, Jaskiran Kaur, Nicole Winch and Anjali Behal from the Surveillance Systems and Data Management Division at PHAC for their contributions to the management of CYP-C and their thorough understanding of the data. As well, we would like to acknowledge Owen Wesley Smith-Lpine from the same division, who is responsible for the development and maintenance of the CYP-C data tool hosted on Health Infobase. Lastly, we would like to acknowledge Paul Gibson (McMaster Children’s Hospital; POGO), Randy Barber (C^17^ Council) and Miranda Fidler-Benaoudia (Alberta Health Services), whose knowledge and expertise of the issues experienced by children and youth with cancer in Canada helped guide the analysis of the data included in the CYP-C data tool.

## Funding

None.

## Conflicts of interest

None.

## Authors’ contributions and statement

KM: Conceptualization, formal analysis, methodology, writing—original draft.

LX: Conceptualization, formal analysis, methodology, writing—review and editing.

RK: Conceptualization, methodology, validation, writing—original draft.

CR: Conceptualization, formal analysis, methodology, writing—review and editing.

VG: Conceptualization, methodology, writing—review and editing.

All authors approved the final paper.

The content and views expressed in this article are those of the authors and do not necessarily reflect those of the Government of Canada.
